# From Mesenchymal Stromal/Stem Cells to Insulin-Producing Cells: Progress and Challenges

**DOI:** 10.1007/s12015-020-10036-3

**Published:** 2020-09-03

**Authors:** Mohamed A. Ghoneim, Ayman F. Refaie, Batoul L. Elbassiouny, Mahmoud M. Gabr, Mahmoud M. Zakaria

**Affiliations:** Urology and Nephrology Center, Mansoura, Egypt

**Keywords:** MSCs, differentiation, IPCs, transplantation, encapsulation, scaffolds, immunomodulation, diabetes

## Abstract

Mesenchymal stromal cells (MSCs) are an attractive option for cell therapy for type 1 diabetes mellitus (DM). These cells can be obtained from many sources, but bone marrow and adipose tissue are the most studied. MSCs have distinct advantages since they are nonteratogenic, nonimmunogenic and have immunomodulatory functions. Insulin-producing cells (IPCs) can be generated from MSCs by gene transfection, gene editing or directed differentiation. For directed differentiation, MSCs are usually cultured in a glucose-rich medium with various growth and activation factors. The resulting IPCs can control chemically-induced diabetes in immune-deficient mice. These findings are comparable to those obtained from pluripotent cells. PD-L_1_ and PD-L_2_ expression by MSCs is upregulated under inflammatory conditions. Immunomodulation occurs due to the interaction between these ligands and PD-1 receptors on T lymphocytes. If this function is maintained after differentiation, life-long immunosuppression or encapsulation could be avoided. In the clinical setting, two sites can be used for transplantation of IPCs: the subcutaneous tissue and the omentum. A 2-stage procedure is required for the former and a laparoscopic procedure for the latter. For either site, cells should be transplanted within a scaffold, preferably one from fibrin. Several questions remain unanswered. Will the transplanted cells be affected by the antibodies involved in the pathogenesis of type 1 DM? What is the functional longevity of these cells following their transplantation? These issues have to be addressed before clinical translation is attempted.

**Graphical Abstract:**
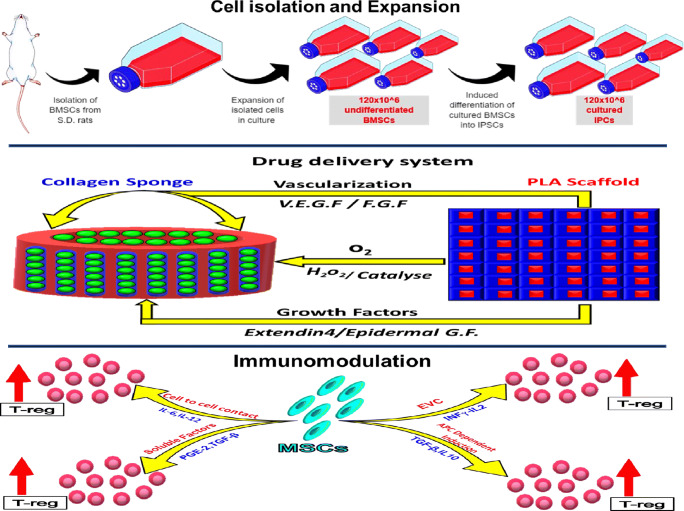
Bone marrow MSCs are isolated from the long bone of SD rats. Then they are expanded and through directed differentiation insulin-producing cells are formed. The differentiated cells are loaded onto a collagen scaffold. If one-stage transplantation is planned, a drug delivery system must be incorporated to ensure immediate oxygenation, promote vascularization and provide some growth factors. Some mechanisms involved in the immunomodulatory function of MSCs. These are implemented either by cell to cell contact or by the release of soluble factors. Collectively, these pathways results in an increase in T-regulatory cells.

Bone marrow MSCs are isolated from the long bone of SD rats. Then they are expanded and through directed differentiation insulin-producing cells are formed. The differentiated cells are loaded onto a collagen scaffold. If one-stage transplantation is planned, a drug delivery system must be incorporated to ensure immediate oxygenation, promote vascularization and provide some growth factors. Some mechanisms involved in the immunomodulatory function of MSCs. These are implemented either by cell to cell contact or by the release of soluble factors. Collectively, these pathways results in an increase in T-regulatory cells.

## Introduction

Diabetes mellitus (DM) is a major health concern. In 2014, more than 400 million people suffered from DM globally compared to 108 million in 1980. If this trend continues, the number is expected to increase to more than 600 million by 2045 [1]. Type 1 DM (T1DM) accounts for ≈5% of all diabetic patients and is the result of destruction of pancreatic islets through an autoimmune-mediated response. Patients depend on exogenous insulin injections throughout their life. However, this treatment does not prevent acute or chronic complications. Glycemic control without a need for exogenous insulin can be achieved by β-cell replacement through transplantation of the whole pancreas or its islets. Despite the increasing success of both approaches, their applications are limited by organ availability and the need for life-long immunosuppression.

Recent progress in the field of regenerative therapies provides an alternative means through the generation of surrogate β-cells from various stem cell sources. Soria and associates reported successful production of insulin-secreting cells derived from embryonic stem cells (ESCs) by gene transfection of a human insulin gene in mice [2]. Lumelsky et al. reported successful differentiation of mouse ESCs using a five-step protocol [3]. The Lumelsky protocol was then modified by Segev et al. by adding a suspension culture step at the end of the differentiation scheme [4]. These early reports were challenged by Rajagopal and colleagues, who argued that the presence of insulin in the cells is due to its sequestration from the culture media and not from intrinsic synthesis [5]. In a landmark study, Kubo and associates reported the development of definitive endoderm from ESCs in culture using activin A [6]. This finding was successfully reproduced one year later by D’Amour et al. [7]. On this basis, protocols for directed differentiation of human ESCs (hESCs) towards pancreatic endocrine lineage were developed [8, 9]. These cells were believed to undergo further maturation following their transplantation under the influence of the in vivo milieu. Rezania and colleagues developed a 7-step protocol that converts hESCs into insulin-producing cells (IPCs) that could reverse diabetes in mice 40 days after transplantation [10]. A multistep differentiation protocol for hESCs or human induced pluripotent stem cells (hiPSCs) was developed by Pagliuca et al. [11]. The aim of this study was to generate glucose-responsive mature β-cells at the end of in vitro differentiation. However, the use of human pluripotent stem cells to generate IPCs has 2 major drawbacks: immunogenicity and teratogenicity. These 2 issues dictate the need for encapsulation of these cells within an immunoisolation device for transplantation in a clinical setting.

A viable alternative can be provided by mesenchymal stem/stromal cells (MSCs). These cells are widely available in many tissues, and can be readily expanded in vitro with a doubling time of 48-72 hours [12]. Many studies have reported that MSCs can differentiate into mesodermal and non-mesodermal lineages [13]. In most animal studies and clinical trials, MSCs were reported safe and well tolerated. Compared to ESCs and iPSCs, human MSCs (hMSCs) were found to impose a negligible teratogenic risk. However, their possible role in the spread and metastasis of pre-existing cancer was noted [14]. MSCs also lack the expression of HLA class II antigens and have a possible immunomodulatory function. These characteristics may, therefore, allow their application in an allogenic setting. Thus, MSCs have become a promising therapeutic agent in regenerative medicine and are the subject of intensive research [15]. An appraisal of the use of MSCs to produce IPCs is timely and is the subject matter of this review. Studies on islet transplantation or pluripotent stem cell-derived β cells are frequently quoted to note their shortcomings and exploit any advantages.

## Mesenchymal Stem/Stromal Cells

### Nomenclature

Previous studies by Friedenstein et al. revealed that cells from bone marrow stroma could generate bone, fat and cartilage cells following heterotopic transplantation [16]. These results suggested the existence of non-haematopoietic bone marrow precursor cells with skeletal and adipogenic potential. For these cells, the term stromal cells was first suggested by Owen [17]. The term mesenchymal stem cells was then popularized by Caplan to refer to plastic-adhering cell preparations isolated from various tissues [18]. More recently, leading investigators of mesenchymal cell therapy have provided convincing data showing that the “stemness” of these cells is lacking [19]. Since these cells are found within the supportive stroma of their resident tissues, the term mesenchymal stromal cell was introduced, which allows the abbreviation “MSCs” to be unchanged. Given the multipotent differentiation capacity of these cells, they have been finally named multipotent mesenchymal stromal cells by the International Society for Cellular Therapy [20]. The term mesenchymal was maintained to indicate the origin and not the differentiation capacity of these cells.

### Characteristics

The defining features of MSCs are inconsistent. Many laboratories have developed methods to isolate and expand MSCs, which can result in subtle differences. To address this issue, the Mesenchymal and Tissue Stem Cell Committee proposed a set of standards to define human MSCs [21]. First, MSCs must be plastic adherent when maintained in standard culture flasks. Additionally, ≥95% of the MSCs must express CD105, CD73, and CD90 and lack expression (<2%) of CD45, CD34, CD14 and HLA class II antigens as shown by flow cytometry. Finally, these cells must be able to differentiate into osteoblasts, adipocytes and chondroblasts under standard in vitro differentiating regimens. These characteristic features are subject to changes as a result of several variables, such as culture conditions, tissue of origin, number of population doublings and passage density [22]. Hence, additional markers, including stro-1, CD271, SSEA-4, and CD146, were suggested for more specific identification [23]. MSCs can also express HLA-DR class II antigens under stimulatory conditions, such as with interferon Ɣ. Furthermore, after 22 population doublings, these cells were reported to lose their adipogenic potential [24]. These factors must be considered when MSCs are used as such or after their differentiation towards a specific lineage.

### Variations on a Theme

Different culture conditions for MSCs have important implications for the final cell population, as they may selectively support the expansion of a certain subpopulation [22]. Reyes and associates described a culture system for MSCs that favors the selection of a subpopulation of primitive cells referred to as multipotent adult progenitor cells or MAPCs [25]. Later, these authors had to retract their publication due to lack of reproducibility and contradictory findings by other investigators [26]. Kogler et al. described a pluripotent CD45(-) cell population from human cord blood [27]. This population shows adherent growth and can be expanded without losing pluripotency. These cells were termed unrestricted somatic stem cells or USSCs. D’Ippolito et al. reported the isolation of a population of pluripotent human cells from bone marrow after their expansion in media similar to that in the in vivo microenvironment of the most primitive cells. These cells were called marrow-isolated adult multilineage inducible or MIAMI cells [28]. Kucia et al. identified a population of CXCR4(+), very small embryonic-like stem cells (VSELs) in murine bone marrow and human cord blood [29]. A population of CD105(+) and SEEAA-3(+) cells was isolated from bone marrow stromal cells by Kuroda and coworkers. This population was defined as multilineage stress enduring (Muse) cells [30].

On a different note, the relationship between MSCs and pericytes (Rouget cells) is unclear. Studies have shown that MSCs are localized next to blood vessels [31]. Pericytes also have a perivascular location and display a marker profile and multipotent differentiation potential similar to MSCs [32]. Thus, distinguishing between the two cell types is difficult. Two alternative explanations were proposed: either pericytes are MSCs with a perivascular location or pericytes represent a distinct subpopulation of MSCs [33]. Pericytes are identified by surface marker expression of NG2 and CD146. In culture, CD146(+) MSCs form endothelial tube-like structures, while CD146(-) cells do not. However, CD146(-) MSCs were shown to acquire a CD146(+) phenotype in culture [34]. On this basis, MSCs can only be distinguished from pericytes by their lack of angiogenic function [32].

MSCs are a heterogeneous population of multipotent stromal cells. Attempts to isolate different subpopulations are work intensive and could be confusing. In this review, the specific features identified by the International Society for Cellular Therapy will be used.

### Sources

For differentiation into IPCs, MSCs were derived from both perinatal and adult tissues. Perinatal sources include the amniotic fluid [35], Wharton’s jelly [36], umbilical cord stroma [37, 38], umbilical cord blood [39] and placenta [40]. Adult sources include bone marrow [41–46], adipose tissue [47–49], dental pulp [50, 51], tonsils [52], endometrium [53], periosteum [54], peripheral blood [55], and liver cells [56]. Among these sources, bone marrow and adipose tissue are the most studied. While obtaining a sample from the bone marrow is a painful invasive procedure, liposuction aspiration is frequently practiced, and their plentiful yield should not be wasted. The volume of human marrow retrieved under local anaesthesia does not exceed ≈40 ml. In contrast, a harvest from adipose tissue under local anaesthesia is ≈200 ml. According to Strem et al., 1 ml of adipose tissue aspirate yields ≈5000 adipose tissue mesenchymal stem cells (AT-MSCs). A similar volume of bone marrow aspirate yields approximately 600-1000 bone marrow mesenchymal stem cells (BM-MSCs) [57]. These data indicate a clear advantage of adipose tissue as a source of MSCs. One possible application for bone marrow is the use of BM-MSCs in autologous protocols. Similarly, stored umbilical cord-derived MSCs can serve as an excellent autologous source for their donors in case of a future need. Recently, there is an increasing interest in the use of Wharton’s jelly-derived MSCs to generate IPCs [58, 59]. It provides an unlimited source for MSCs without ethical considerations. One million MSCs can be collected from a 20-cm umbilical cord. In addition, the derived MSCs have a high replication capacity without observed senescence up-to 80 population doublings [60].

### Isolation, Expansion and Verification

Isolation of MSCs from tissue samples relies on their adherence to the plastic material of the culture plates. Before sampling, prospective donors must be screened for communicable disease. In addition, a biochemical and hematological profile is obtained with special emphasis on a coagulation profile. The methods of isolation and expansion of MSCs from bone marrow or liposuction aspirates were described in detail by Gabr et al. [61]. Modified protocols for isolation and expansion were devised to enhance the yield, and hence, clinical applicability was also reported [62, 63]. The purity of isolated and expanded MSCs must be tested according to the criteria specified by the International Society for Cellular Therapy [21].

## From MSCs to IPCs

In general, IPCs could be derived from MSCs by one of two approaches: gene manipulation or directed differentiation. For analysis of successful production of surrogate β-cells, seven criteria were suggested by Calne and Ghoneim [64] and are as follows: 1. Presence of insulin storage granules in the surrogate β-cells. 2. Co-expression of C-peptide and insulin in the same cells. 3. Release of insulin and C-peptide in response to a glucose challenge. 4. Control of hyperglycemia following transplantation. 5. Weight gain and a responsive glucose tolerance curve of the transplanted animals. 6. Prompt return of diabetes when the surrogate β-cells are removed. 7. No regeneration of islets observed among the chemically induced diabetes in animals or regeneration of pancreas in the pancreatectomized animals.

### Gene Manipulation

This procedure can be implemented by either gene transfection using a viral vector or by genetic engineering.

#### Genetic Transfection

Gene transfection can be carried out either by in vitro gene delivery into cells, which are then transplanted into a recipient, or by direct delivery of genes in vivo. Chen and associates transfected liver cells in vitro with a plasmid harboring the human insulin gene [65]. The resulting cells were injected back as an autologous graft in the liver of diabetic pigs. According to these authors, insulin production and improvement of hyperglycemia were observed for more than 9 months. Direct infusion of a lentiviral vector encoding a human gene into the portal system of rats with streptozotocin (STZ)-induced diabetes was reported by Ren et al. [66]. The blood glucose of the treated animals was normalized for 500 days. Karnieli et al. transfected human bone marrow MSCs with rat *Pdx1* using a BABE-hygromycin vector [67]. Although glucose-stimulated insulin secretion was demonstrated in vitro, the cells lacked *Neurod1* expression. Transplantation of these cells in mice with STZ-induced diabetes resulted in their further differentiation with expression of *Neurod1* and a reduction of hyperglycemia. Qing-Song and coworkers transfected MSCs from murine bone marrow with 3 transcription factors, *Pdx1*, *Neurod1* and *Mafa,* using an adenoviral vector [68]. The transfected cells were then transplanted into the liver parenchyma of mice with chemically induced diabetes. Seven days after transplantation, the treated animals demonstrated glucose tolerance curves similar to the normal controls. However, this result was not sustained after 14 days, presumably due to unstable or transient gene expression. Boroujeni and Aleyasin transfected human AT-MSCs (hAT-MSCs) with *PDX1* using a lentivirus vector [69]. The transfected cells were then cultured in high-glucose DMEM supplemented with B27, nicotinamide and fibroblast growth factor. The expression of *PDX-1, NGN3 and GLUT2* was detected by RT-PCR. Four million cells were intraperitoneally transplanted into Sprague-Dawley rats with alloxan-induced diabetes. The authors reported that hyperglycemia was normalized within 3-4 days and maintained for several months, which was astonishing since it followed a xenogeneic transplantation without immunoisolation or immunosuppression. Thi Do and associates transfected porcine bone marrow-derived MSCs with the insulin gene using a lentiviral vector [70]. Autologous transplantation of the treated cells in the liver of pigs with STZ-induced diabetes resulted in partial improvement of their hyperglycemia. The generation of IPCs from hBM-MSCs by transfection with both miR-375 and anti-miR-9 was reported by Jafarian and associates [71]. The authors suggested that while miR-375 is responsible for insulin gene expression and secretion, miR-9 inhibits insulin exocytosis. Bai et al. generated IPCs from nestin-positive umbilical cord MSCs of chickens by transfection with miR-375 and miR-26a [72]. These cells were then transplanted under the renal capsules of SCID mice with chemically induced diabetes. Two weeks after transplantation, chicken insulin was detected in the sera of glucose-challenged mice.

Although MSCs were not involved in their experiments, a brief account of the experimental findings of Fatima Bosch and her group from Barcelona is worth mentioning. In 2006, this group reported successful treatment of mice with STZ-induced diabetes by intramuscular injection of an adeno-associated vector (AAV) encoding the genes for insulin and glucokinase [73]. Insulin production in addition to glucose phosphorylation were necessary to achieve normoglycemia. In 2013, this group published the results of treatment of chemically induced diabetes in 4 dogs using the same principle: intramuscular injection of an AAV vector encoding the insulin and glucokinase genes [74]. Normalization of fasting glucose and accelerated normoglycemia after a glucose challenge without episodes of hypoglycemia were noted. This benefit was maintained for 4 years. In a follow-up study, the authors reported that normoglycemia in 2 of the treated dogs was sustained for 8 years [75]. We question why an approach with such excellent results was not translated to the clinic or whether these observations were the result of regeneration of the native pancreata.

#### Gene Editing

The use of viral vectors has major limitations due to possible oncogene transactivation and the lack of physiological expression that allows monitoring. Recently, gene therapy researchers have focused on gene editing technologies as an alternative approach [76]. The breakthrough in genome editing started in 2013, when the first CRISPR/Cas9 system was engineered to work in mammalian cells [77, 78]. The endonuclease activity of Cas9 can be inactivated, forming nuclease-deficient Cas9 (dCas9). Transcriptional activators can be fused to dCas9. Subsequently, this combination can be guided by a target-specific RNA (sgRNA) to the upstream promotor region of an endogenous gene, leading to the upregulation of its expression [79]. If a single sgRNA is used, activation of a given gene is negligible. Multiplexing with 3 or more sgRNAs leads to synergistic activation with a significant increase in gene expression [80]. Gimenez et al. successfully induced endogenous human insulin transcription using the dCas9-VP160 transcriptional activator and multiple insulin promoter targeting RNAs in human embryonic kidney cells and human fibroblasts [81]. The CRISPR/Cas9 gene editing system was also used to identify the role of several transcription factors involved in pancreatic embryonic development [82]. These investigators generated mutant lines affecting 8 transcription factors. Six of these genes *(PDX1, RFX6, PTFIA, GLIS3, MNX1 and NGN3)* were found to be associated with permanent neonatal diabetes mellitus (PNDM). Mutations in a subset of these genes (*PDX1 and PTF1A*) are associated with several deficiencies in endocrine and exocrine functions (pancreatic agenesis). Induced pluripotent stem cells were generated from skin fibroblasts of a patient with PNDM followed by their differentiation into the pancreatic endocrine lineage. The resulting cells did not contain or secrete insulin. Genetic editing of these cells using the CRISPR/Cas9 system was carried out. A guide RNA against the insulin locus close to the mutation site was designed along with a correction template. Genetically corrected cells showed ≈53% insulin-positive cells [83]. Collectively, the field of genetic engineering is rapidly evolving. We should investigate further developments and refinements that may lead to possible clinical applications.

### Directed Differentiation

Several authors have reported that under certain culture conditions, MSCs can form cells that do not belong to a mesodermal lineage [84–86]. This phenomenon can reflect trans-differentiation or differentiation of a pluripotent subgroup of a heterogeneous population [15]. In 2004, 3 groups of investigators reported successful differentiation of murine bone marrow-derived MSCs into IPCs [87–89]. These early observations were reproduced by Timper and her group using hAT-MSCs [47]. Sun and associates could differentiate hBM-MSCs from diabetic patients to form IPCs [41]. These early observations were followed by multiple reports using either BM-MSCs or AT-MSCs [43, 45, 48, 90–92]. To this end, various protocols were employed. In general, cells were cultured in glucose-rich media with different growth and activation factors. In our laboratory, hBM-MSCs were obtained from diabetic as well as healthy volunteers, and a 3-step differentiation protocol was used [42]. Initially, mercaptoethanol was used to induce *PDX1* expression in the cells. Subsequently, nonessential amino acids, basic fibroblast growth factor, epidermal growth factor and B27 supplement were added. Finally, activin A and nicotinamide were supplemented. At the end of differentiation, ≈5% of the cells tested positive for insulin and C-peptide. This modest yield did not vary between cells obtained from diabetic or healthy individuals. Electron microscopy with nanogold immunolabelling revealed C-peptide granules at the rough endoplasmic reticulum. A stepwise increase in the release of insulin and C-peptide in response to increasing glucose concentrations was also noted. Moreover, the differentiated cells expressed all the relevant pancreatic endocrine genes. Transplantation of these cells under the renal capsule of nude mice with STZ-induced diabetes resulted in the control of their diabetes within 7-10 days. The sera of the treated animals contained human insulin and C-peptide, with negligible levels of mouse insulin. When the cell-bearing kidney was removed, diabetes rapidly returned. In other words, the 7 criteria were satisfied in this experimental trial [64]. In another study, the yield of IPCs from hBM-MSCs and hAT-MSCs was compared. The results were essentially the same without a significant difference [61]. The relative efficiency of 3 differentiation protocols was also evaluated [93]. The yield of functional IPCs was modest and comparable among the 3 methods. Given its simplicity and the short duration required for its completion, trichostatin-A (TSA)/glucagon-like peptide-1 (GLP-1), introduced by Tayaramma [94], became our method of choice. Despite these modest results, we demonstrated that after transplantation, the proportion of differentiated cells increased to reach ≈18%, presumably due to favorable in vivo factors [95]. Efforts to improve the yield of IPCs using various strategies, including differentiation in a suspension culture [44], on a scaffold [96] or within an extracellular matrix, were reported [97]. Choi et al. [98] and Xie et al. [99] observed that an extract from an injured pancreas could promote the differentiation of rat MSCs into IPCs. In a proteomic study, 3 proteins were differentially expressed from injured pancreata of Sprague-Dawley rats: cofilin1, nucleoside diphosphate kinase (NDPKA) and peroxiredoxin-6 (PRDX6) [100]. The yield of IPCs when these proteins were added to the differentiation medium alone or in combination was evaluated [101]. The best outcome was observed in samples supplemented with PRDX6 alone, where the yield of IPCs increased by 4-fold compared to that of the controls.

The results of cell therapy for experimental type 1 DM from 3 leading groups were compiled by Schulz [10]. Table 1 is a summary of these data. The results of MSC-derived IPCs from 2 studies were also added for comparison [42, 45]. The data shows that the efficiency of MSCs as a source for IPCs is as good as, if not better than, pluripotent stem cells.

**Table 1:** Comparison of experimental cell therapies for type 1 DM

Variable	Keiffer (10)	Melton (11)	Kroon (8) Schulz (9)	Ghoneim (42)	Xin (45)
Source of Cells	ESCs	ESCs, iPSCs	ESCs	MSCs	MSCs
Cellular Product	β cells	β cells	Pancreatic progenitor cells	IPCs	IPCs
Differentiation Scheme	7 steps	6 steps	4 steps	3 steps	3 steps
Length of Differentiation (days)	27-42	27-35	12	22	29
Implant Dose	1.25 x 10^6^	5 x 10^6^	3 x 10^6^	3 x 10^6^	3 x 10^6^
Site of Implant	Kidney capsule	Kidney capsule	Subcutaneous	Kidney capsule	Kidney capsule
Time to Correct Hyperglycaemia in Mice (days)	≈ 50-60	≈ 75	≈ 50-70	≈ 7-10	6
Clinical Trial	----	-----	Yes (Phase I/II)	-----	-----

## Selection of a Site for Cell Transplantation

The sites for cell transplantation were summarized by several thorough reviews based on studies of islet transplantation [102–105]. A number of factors can influence the choice of a suitable site: an animal experiment or a clinical application? A small animal or a large one? Autologous cells or allogenic cells? Transplantation of free cells, cells on a scaffold or cells in an encapsulation device? For animal experiments, cells were grafted under the skin [106], in striated muscles [107], within the epidydimal fat pad [9], under the renal capsules [42] or inside an omental pouch [108], among several other sites. For a clinical application, choices are more restricted. When IPCs derived from MSCs are considered, two sites provide a distinct advantage: under the skin or within an omental pouch. The subcutaneous sites are easily accessible, and a minimally invasive procedure can be used. The main disadvantage of these sites is the poor blood supply. To overcome this problem, Pepper et al. suggested a 2-stage procedure [106]. In the first stage, plastic tubes were inserted under the skin to induce the formation of new blood vessels. Cell transplantation into this pre-vascularized bed was then carried out in a second stage. In contrast, the omentum has a rich vascular supply in which capillaries form numerous spiral loops. This capillary bed lies directly under a very thin layer of mesothelium. Thus, early oxygenation for the transplanted cells is guaranteed with free exchange of glucose, insulin and metabolites. Furthermore, omental venous blood drains into the portal system. As early as 1983, Yasunami and associates reported experimental islet transplantation in a peritoneal-omental pouch [108]. Evidence for the superiority of the omentum over other sites for cell engraftment and function was later documented in 2 experimental studies [109, 110]. The feasibility of creating an omental pouch suitable for clinical application was explored by Berman et al. [111]. Diabetes was chemically induced in rats as well as non-human primates. Islets were implanted onto the omentum within a plasma-thrombin biological scaffold. The study concluded that the feasibility and efficiency of this protocol justified proceeding to a pilot phase I/II clinical trial. To our knowledge, the first clinical trial was published as a case report by Baidal and colleagues [112]. This trial is a part of an ongoing study entitled “Allogenic islet cells transplanted onto the omentum” (ClinicalTrials.gov. Identifier: NCT 02213003). A 43-year-old female patient with a 25-year history of type I DM was controlled by exogenous insulin. Allogenic islets were transplanted laparoscopically on the omentum in a fibrin scaffold generated from autologous plasma and recombinant thrombin. The patient received induction followed by maintenance immunosuppression. Insulin was discontinued 17 days after transplantation. With continuous glucose monitoring, the 7-day mean glucose level was ≈109 mg/dL, and the glycated haemoglobin was 6.0%. The patient exercised regularly and followed a low-carbohydrate diet, which probably contributed to her stable glycemic control. At 12 months, a functional decline was noted. The authors speculate that this finding might be due to a switch from tacrolimus to sirolimus for maintenance immunosuppression. Nevertheless, the patient continued to have stable control without exogenous insulin or hypoglycemic episodes. These encouraging results will pave the way for many clinical trials using different sources of IPCs. If IPCs derived from MSCs are used, immunosuppression may not be necessary, avoiding its possible undesirable side effects.

## Platforms for Cell Transplantation

IPCs, generated from various sources, can be engrafted as free cells. This procedure is usually performed under certain experimental conditions, such as transplantation under the renal capsules of immune deficient mice. Alternatively, cells may be encapsulated within an immunoisolation device or on a scaffold material.

Immunoisolation is necessary to protect the graft from allogenic responses and/or autoantibodies. This condition is usually achieved by cell encapsulation within a biocompatible semipermeable membrane**.** Cell transplantation in encapsulation devices has several challenges and should meet certain requirements. The permeability of such a membrane should allow the free exchange of oxygen and nutrients with good insulin kinetics in response to changes in blood glucose levels. Moreover, the membrane should block high molecular weight complexes, cytokines and immune cells. Early oxygenation of the enclosed cells must be ensured, and the host responses in the form of a foreign body tissue reaction should be minimized to prevent pericapsular fibrosis. Immunoisolation devices are classified according to their size into microencapsulation or macroencapsulation.

### Microencapsulation

Lim and Sun were the first to report microencapsulation for islet transplantation [113]. Since then, a growing number of studies in rodents, pigs, dogs and non-human primates have been conducted, employing a wide range of materials [114]. Typically, cells are incorporated in an alginate hydrogel covered with a semipermeable membrane (usually poly-L-lysine or ornithine) to provide appropriate permeability and mechanical strength. While these capsules can support islet function in small animals, they were inefficient in those with strong immune systems, including large animals, non-human primates and humans [115]. In 1994, Soon-Shiong et al. reported the first clinical trial of microencapsulated intraperitoneal islet transplantation in a type I diabetic patient. These investigators reported early insulin independence [116]. However, the patient had to receive exogenous insulin followed by a second transplant to maintain glycaemic control for 58 months.

Recently, several attempts to improve the functional longevity of microencapsulation were reported. Dang and associates showed that alginates mixed with curcumin inhibit foreign body reactions when transplanted under the skin [117]. Hybrid alginate microcapsules incorporating curcumin were used to encapsulate pancreatic islets derived from Sprague-Dawley rats. When transplanted in mice with STZ-induced diabetes, they could provide better control of blood sugar than that of the controls for 2 months. The microencapsulation device was optimized by conformal coatings [118]. The aim was to conform the coating of the microcapsules according to the shape and size of the encapsulated cells. A coating with a uniform thickness was obtained, in contrast to conventional methods in which capsules of a similar diameter are engineered independent of the size of enclosed cells. Veiseh and associates studied the influence of the size of the microcapsules on the intensity of the foreign body tissue reaction and/or the induction of an immune response [119]. They concluded that larger alginate spheres in the range of 1.5 mm reduced fibrosis and minimized immune cell deposition. They also reported that when rat islet cells encapsulated in 1.5 mm alginate capsules were transplanted in mice with STZ-induced diabetes, blood glucose levels were controlled 5 times longer than that with conventionally sized 0.5 mm capsules. Materials that mitigate foreign body reactions in primates were studied by Vegas et al. [120]. These researchers identified 3 triazole analogues that substantially reduced the foreign body tissue reaction, recognition by macrophages and fibrosis. In a subsequent study, the same group reported that triazole-thiomorpholine dioxide (TMTD) alginate capsules incorporating mature β-cells derived from hESCs provided long-term glycemic control without immunosuppression when transplanted in immune-competent mice [121].

### Macroencapsulation

These are further classified into either intravascular or extravascular devices. Intravascular macroencapsulation involves loading of the engrafted cells within hollow semipermeable tubes that are directly connected to the recipient vasculature. The transplanted cells are in direct contact with the bloodstream and receive an adequate supply of oxygen and nutrients. However, this method is associated with several limiting complications, such as clot formation and embolization [122]. Extravascular macroencapsulation can be engineered in a tubular or a planar configuration. Tubular devices are weak and susceptible to rupture, whereas planar devices are more stable. In addition, planar devices can be retrieved at predetermined time points for examination of their content. This flat design allows a suitable cell seeding density relative to the surface area. Nevertheless, macroencapsulation devices face a number of important challenges. The enclosed cells have to be near a blood supply at a distance not exceeding 150–200 μm to allow diffusion of oxygen and nutrients. Cells located away from an adequate blood supply are subject to necrosis and death. Furthermore, since no direct vascular access is present, the diffusion time for oxygen, glucose and insulin is prolonged. Thus, the production of insulin and its release are delayed. Accumulation of insulin within the capsule puts the enclosed graft at risk of insulin inhibition from their own product [123]. Data from clinical islet transplantation indicate that ≈5000 islet equivalents per kg body weight (IEQ/kg) is required for the control of diabetes. For a 70-kg person, ≈350,000 IEQ will be required. The surface area to volume ratio must be optimized to minimize the diffusion distances between the host vasculature and the encapsulated cells. It is suggested that a surface area of ≈460 cm^2^ is needed if 350,000 IEQ are to be encapsulated [124]. To overcome the problem of cell hypoxia, researchers used several approaches. A 2-stage procedure was reported, where subcutaneous implantation of an empty device was initially performed to induce vascularization. In the second stage, cells were loaded into the pre-vascularized encapsulation device [125, 126]. Alternatively, a biological oxygen donor may be used to support in situ oxygen requirements and prevent hypoxia-induced cell damage [127, 128]. Another group of investigations found that the incorporation of vasculogenic agents enhances the survival and function of encapsulated cells [129–131]. An interesting new material, HEMOXCell, is an extracellular haemoglobin extracted from a marine invertebrate (Hemarina, Morlaix, France). This substance is nonimmunogenic and can bind to 156 oxygen molecules (human haemoglobin can bind a maximum of 4), and oxygen is delivered on demand. When added to MSCs in vitro cultures, HEMOXCell increased the cell proliferation rate and preserved their viability [132]. The efficiency of HEMOXCell and perfluorodecalin as biological carriers was compared. Both agents were added to in vitro cultures of islets derived from Wistar rats. Under hypoxic conditions, the use of HEMOXCell maintained islet viability and restored its function, while perfluorodecalin did not [133].

The TheraCyte capsule is a bilayer planar device that was used in several experimental studies (Irvine, CA, USA). This device consists of an outer layer of polytetrafluoroethylene (PTFE) with 5 μm sized pores. The inner layer is made from the same material with a pore size of 0.45 μm to provide immunoisolation. A polyester mesh is attached to the outer layer to induce vascularization. In several studies, this device was shown to provide immunoisolation in allogenic and xenogeneic settings. Variable degrees of control of diabetes were also reported [133–137]. In a preliminary study, Gabr et al. differentiated hBM-MSCs to form IPCs. The differentiated cells were loaded into TheraCyte capsules and transplanted into dogs with chemically induced diabetes at a dose of 5x10^6^ cells/kg. Four of 6 dogs became euglycemic for a period of 6 months. Thereafter, there was a gradual and progressive rise in blood glucose levels [138]. Histology of the explanted capsules revealed a significant amount of pericapsular fibrosis. In another study, pancreatic progenitor cells derived from hESCs were transplanted in the epididymal fat pads or under the skin within a TheraCyte capsule in athymic nude rats [139]. The authors reported that human C-peptide and insulin were detected at very low levels without an increase following a glucose challenge. These results indicate that the extent of endocrine cell formation or secretory function does not qualify for clinical application.

A modified TheraCyte capsule, Encaptra, was developed by Viacyte (Viacyte Inc., San Diego, CA). The device was loaded with pancreatic progenitor cells derived from hESCs [8]. Evidence has shown that transplantation of this combination can control chemically induced diabetes in rodents [9, 140]. On behalf of Viacyte, the results of the first clinical trial using this system for islet replacement in type I diabetic patients were presented by D’Amour, in the 2018’s International Pancreas and Islet Transplantation Association meeting, and reported by Odorico et al. [141]. The study included 19 patients in an open-label trial. A high degree of patient variability was observed. The 12-week explants showed minimal cell survival with cell death likely due to hypoxia. The trial was paused to improve their immunoisolation device. In a second attempt, their cells are to be encapsulated in an open system. Thus, anti-inflammatory agents as well as standard immune suppression have to be administered.

In another study, Pepper and associates developed what is known as the Sernova pouch (Sernova Corp., London, Ontario, Canada). This device is macroporous and was not intended to be immunoisolating. The empty device was inserted under the skin of BALB/c mice to provoke neovascularization. Four weeks later, chemically induced diabetes was induced, and syngeneic islets were transplanted into the pouch. To serve as a control, another group of animals was transplanted with islets under the renal capsule. Diabetic control was comparable between the groups [142]. The authors concluded that this device is biocompatible and provides a suitable environment for islet transplantation. However, a 3-year phase I/II clinical study using this device was terminated after recruiting 3 patients [143]. This finding strongly shows that translation from experimental findings to a clinical trial is not always successful.

Ludwig and associates developed a macroencapsulation device under the commercial name β-Air. This device consists of 3 compartments layered in a disc-shaped capsule. The outer 2 compartments house the transplanted tissue, while the middle compartment serves as an oxygen reservoir that can be refilled from an external oxygen delivery system via an access port. A pilot clinical study was carried out in which the β-Air device containing 2100 IEQ/kg was transplanted preperitoneally in a 63-year-old male patient with a long history of treatment for type 1 DM. During a follow-up period of 10 months, persistently low HbA1c with a reduction in insulin requirements was observed [144]. Despite this modest result, a clinical trial with human islets was initiated (ClinicalTrials.gov.Identifier:NCT02064309). Nevertheless, Korsgren reported that the kinetics of insulin release from β Air are far from ideal [145]. Maximal insulin secretion following a glucose challenge occurred after ≈4 hours. In contrast, maximal insulin secretion from non-encapsulated (native) islets occurs within a few minutes. Carlsson and associates reported their clinical experience with the β-Air device in a phase 1 study [146]. Four patients were transplanted with this device into which 1800-4600 IEQ/kg islets were loaded. Patients were followed up for 3-6 months. At the end of the observation period, the implanted devices were retrieved. Improvements in HbA1c were noted in 3 of the 4 patients. However, this improvement was not significant. The insulin requirements were not reduced. Histology of the retrieved capsules demonstrated pericapsular deposition of fibrous tissue. The authors concluded that the device can support the survival of allogenic islets for several months, although the function of the transplanted cells was limited. In our opinion, this conclusion is unsatisfactory, and the reported results do not justify further clinical studies.

It is abundantly clear that the available encapsulation devices require further optimization and refinement. Induction of the foreign body reaction and subsequent fibrosis must be minimized. Adequate vascularization to avoid cell hypoxia must be ensured. Since encapsulation relies on passive diffusion of oxygen, glucose, insulin and metabolites, high blood glucose levels must be reached before insulin is released. Reduction of the distance between the enclosed cells, as much as possible, is also required to minimize the delay in insulin release.

### Scaffolds

Scaffolds are in effect an open system that allows free permeation of oxygen and nutrients. These devices can also allow a 3D arrangement of the loaded cells, a distinct advantage for cell differentiation or transplantation [147–150].

Scaffolds can be engineered from synthetic or natural materials. The different materials utilized for their construction, as well as the advantages and limitations of each, were the subject of extensive reviews [151–153]. Mitrousis and associates outlined the important factors required for the successful use of scaffolds for cell culture and/or cell transplantation [154]. The incorporation of an extracellular matrix (ECM) is necessary to allow cell adhesion and prevent anoikis. Several investigators showed that ECM protein-coated scaffolds promote cell survival and function [155, 156]. Induction of vascularization to provide a supply of oxygen and nutrients must be ensured. Transplantation within a VEGF-containing collagen scaffold significantly increased cell survival and function [157]. The material used for fabrication of a scaffold should evoke no or minimal foreign body reaction. An intense reaction can be detrimental to newly transplanted cells [158]. To this end, Goh et al. suggested the use of a decellularized pancreas as a natural 3D scaffold. After subcutaneous transplantation in mice, no evidence of a foreign body reaction was detected [159]. The shape of the implanted scaffold can also influence the inflammatory response. Mattaga et al. reported that the lowest reaction was elicited when the scaffold has a circular cross section [160]. Again, the engineering of a scaffold should allow even and uniform distribution of the loaded cells. For this purpose, Daoud and associates suggested a pore size of ≈350 μm, a spacing between strands of ≈400 and a strand thickness of ≈100 μm [147].

Two sites are suitable to provide a home for transplanted scaffolds: the subcutaneous tissue and the omentum. Smink et al. evaluated the efficiency of a subcutaneous poly-D,L-lactide-co-ε-caprolactone (PDLLCL) scaffold for islet transplantation [161]. A 2-step procedure was adopted. Initially, the empty scaffold was implanted subcutaneously in nude mice with STZ-induced diabetes to induce vascularization. Four weeks later, rat islets were inserted into the scaffold. Transplantation of 1200 islets controlled diabetes in 100% of the treated animals. Removal of the scaffold was followed by the prompt return of hyperglycemia. The researchers concluded that a pre-vascularized scaffold can maintain the viability and function of subcutaneously transplanted cells. Pedraza and associates fabricated a microporous 3D scaffold from poly(dimethylsiloxane) (PDMS). The efficiency of islets loaded within this scaffold to restore normoglycemia was evaluated in a syngeneic diabetic rat model [162]. The omentum was used as a transplantation site. After the scaffold was placed in the omentum, 1800 IEQ/kg was loaded into the scaffold. The omentum was then wrapped around the scaffold, and the edges were sealed with fibrin gel. Transplantation of free islets in an omental pouch or under the renal capsule served as controls. Transplanted islets within the PMDS scaffold resulted in normoglycemia in 83% of the treated animals. A similar result was obtained with freely transplanted islets, whereas islets transplanted under the renal capsule reversed hyperglycemia in 100% of cases. These results indicated that the omentum is a suitable site to receive freely transplanted cells or cells loaded onto a scaffold. Similar favorable results were reported both experimentally by Berman et al. and clinically by Baidal et al. [111, 112].

In summary, subcutaneous transplantation is minimally invasive and allows retrieval of the implanted scaffolds. Its poor vascularization is a major limitation. This issue can be overcome by a 2-stage transplantation as reported by Smink et al [161]. A one-stage subcutaneous transplantation may also be possible if a drug delivery system is incorporated to provide immediate oxygenation, induce early vascularization and supply necessary growth factors. On the other hand, the vascularity of the omentum is a distinct advantage. Although transplantation into this site involves an invasive procedure, this can be minimized if a laparoscopic intervention is used.

## MSCs and the Immune Responses

An intriguing feature of MSCs is their ability to evade immune recognition and inhibit immune responses [163–165]. MSCs are considered immune privileged since they lack expression of HLA class II antigens and the costimulatory molecules CD40, CD80 and CD86. The potential of MSCs for immunomodulation was recognized more than a decade ago [166, 167]. The mechanisms involved in this immunomodulatory property are diverse and complex and were the subject of detailed reviews [168–170]. This function is evoked under inflammatory conditions and is exerted by the release of soluble factors or through cell-to-cell contact. As a proof of principle, Bartholomew and associates reported one of the first in vitro and in vivo studies [171]. When added to mitogen-stimulated lymphocytes, MSCs resulted in a 50% reduction in their proliferative activity. In vivo, administration of MSCs to mismatched recipient baboons led to prolonged allograft survival of a third-party skin graft.

The immunomodulatory functions of MSCs were explored experimentally in multiple disease models as well as in several clinical trials [172–174]. Lee et al. infused human MSCs in NOD/SCID mice with STZ-induced diabetes. The glycemic levels were reduced, and mouse but not human insulin increased. The pancreatic islets of the treated animals appeared larger, with an increase in insulin mouse reactivity [175]. Madec and coworkers injected MSCs derived from BALB-B mice into 4-week-old NOD female mice to examine the potential benefit of MSCs in cases of spontaneous diabetes [176]. The treatment delayed the onset and decreased the incidence of diabetes in 60% of the treated animals. Yang et al. isolated, expanded and differentiated human umbilical cord MSCs (hUCMSCs) to form IPCs [177]. The resulting IPCs remained hypoimmunogenic and lacked the expression of HLA class II antigens. STZ diabetes was induced in male mice. The diabetic mice were randomly assigned to receive either hUCMSCs or IPCs under the kidney capsule. The blood glucose levels of the IPC-transplanted animals decreased rapidly, while their levels remained unchanged in the hUCMSC-treated group. Thirty days post-transplantation, the removed kidneys from the IPC-transplanted animals showed infiltration by immune cells. The authors suggested that MSCs can become immunogenic after their transplantation as a result of interaction with the disease microenvironment. Changes in the allogenicity of MSCs after their differentiation and/or transplantation were also observed by other investigators [178, 179]. The observed lack of consistent results can be attributed to several additional factors. The tissues from which MSCs were derived were different [180]. The routes of administration were not the same [181]. Moreover, some studies showed that allogenic MSCs are immunogenic and can evoke cell-mediated as well as humoral immune responses [182–184].

These contrasting observations emphasize the need to characterize the intricate details by which MSCs exert immunomodulation. In 2008, del Rio et al. noted the importance of programmed death receptor (PD-1) and its ligands (PD-L_1_, PD-L_2_) in transplantation immunology [185]. Davies and associates reported that MSCs constitutively express PD-L_1_ and PD-L_2_ on their surface [186]. The proinflammatory cytokines INFƔ and TNFα induce upregulation of these ligands. The receptor PD-1 is expressed on the cell surface of activated T and B cells. The interaction between PD-1 and its ligands is accomplished by cell-to-cell contact as well as by the release of soluble factors (sPD-L_1_ and sPD-L_2_). These changes result in abrogation of interleukin-2 (IL-2) secretion, suppression of T cell proliferation, IL-10 production and induction of Tregs. These pathways were confirmed by blocking experiments using anti-PD-L antibodies. Additionally, splenomegaly and increased susceptibility to autoimmune disease were observed in PD-1 knockout mice [187].

The balance between the proinflammatory and immunoregulatory responses following the use of naïve or differentiated MSCs determines the final outcome. If the immunomodulatory functions of MSCs are required, the balance must be tipped towards a net regulatory function. To this end, the expression of PD-L as a biomarker to identify and select low risk-high benefit allogenic cells was recommended [188]. Furthermore, Al-Daccak and Charron suggested that banking of allogenic MSCs can allow the selection of HLA-compatible donors. The size of such a donor bank depends on the frequency of HLA haplotypes. It is estimated that storing ≈100 haplotypes would allow the selection of a compatible donor [189]. It was also advocated that among the suitable allogenic cells, donor-specific antibodies have to be excluded by Luminex-based solid-phase assays [190]. Although these findings are significant, they should not be generalized and have to be carefully verified when IPCs derived from MSCs are considered for clinical application.

## Concluding Remarks

The aim of this review is to identify challenges facing transplantation of hMSC-derived IPCs as a potential cell therapy for type 1 DM. MSCs offer several advantages over other cell sources. Their risk of teratogenicity is negligible. Verification of their immunomodulatory function would prevent the need for life-long immunosuppression or encapsulation within an immunoisolation device. In addition, the efficiency of hMSC-derived IPCs to control chemically induced diabetes in experimental animals is comparable to that of pluripotent stem cell-derived β cells.

MSCs can be obtained from an autologous source. Allogenic hAT-MSCs are a widely available by-product of cosmetic surgeries and should not be wasted. Samples can be stored, and their immunologic identity can be determined to select the optimal donor cells.

For clinical application, 2 sites can provide a suitable home for the transplanted cells: the subcutaneous tissue or the omentum. A 2-stage procedure is required for the former and a laparoscopic intervention for the latter. For either site, cells must be transplanted within a scaffold. Thus far, a biological scaffold from fibrin is adequate for the required goal. In this regard, the initial success of the clinical trial reported by Baidal et al. using the omentum as a transplantation site shows promise [112].

Over the past decade, significant progress has been achieved in the experimental domain. For clinical translation, several issues have to be resolved. Will MSC-derived IPCs retain their immunomodulatory function or become immunogenic? Will they be susceptible to the detrimental effects of existing antibodies that have destroyed the native β cells in type 1 DM? What will be the functional longevity after transplantation? Finally, it must be emphasized that cell therapy for type 1 DM can only be meaningful and clinically justifiable if their functional results are comparable to or better than those of the ever-improving closed-loop insulin pumps.
